# Characterizing offshore polar ocean soundscapes using ecoacoustic intensity and diversity metrics

**DOI:** 10.1098/rsos.231917

**Published:** 2024-08-14

**Authors:** Ramona M. Mattmüller, Karolin Thomisch, Joseph I. Hoffman, Ilse Van Opzeeland

**Affiliations:** ^1^ Department of Evolutionary Population Genetics, Faculty of Biology, Bielefeld University, Bielefeld 33615, Germany; ^2^ Department of Animal Behaviour, Bielefeld University, Bielefeld 33615, Germany; ^3^ Ocean Acoustics Group, Alfred Wegener Institute Helmholtz Centre for Polar and Marine Research (AWI), Bremerhaven 27570, Germany; ^4^ Center for Biotechnology (CeBiTec), Faculty of Biology, Bielefeld University, Bielefeld 33615, Germany; ^5^ British Antarctic Survey, High Cross, Madingley Road, Cambridge, CB3 OET, UK; ^6^ Joint Institute for Individualisation in a Changing Environment (JICE), Bielefeld University and University of Münster, Bielefeld 33615, Germany; ^7^ Helmholtz Institute for Functional Marine Biodiversity (HIFMB), Carl von Ossietzky University Oldenburg, Oldenburg 26129, Germany

**Keywords:** underwater ambient noise levels, one-third-octave-level bands (TOL), MANTA software, passive acoustic monitoring (PAM), sea ice, marine mammals

## Abstract

Polar offshore environments are considered the last pristine soundscapes, but accelerating climate change and increasing human activity threaten their integrity. In order to assess the acoustic state of polar oceans, there is the need to investigate their soundscape characteristics more holistically. We apply a set of 14 ecoacoustic metrics (EAMs) to identify which metrics are best suited to reflect the characteristics of disturbed and naturally intact polar offshore soundscapes. We used two soundscape datasets: (i) the Arctic eastern Fram Strait (FS), which is already impacted by anthropogenic noise, and (ii) the quasi-pristine Antarctic Weddell Sea (WS). Our results show that EAMs when applied in concert can be used to quantitatively assess soundscape variability, enabling the appraisal of marine soundscapes over broad spatiotemporal scales. The tested set of EAMs was able to show that the eastern FS, which is virtually free from sea ice, lacks seasonal soundscape dynamics and exhibits low acoustic complexity owing to year-round wind-mediated sounds and anthropogenic noise. By contrast, the WS exhibits pronounced seasonal soundscape dynamics with greater soundscape heterogeneity driven in large part by the vocal activity of marine mammal communities, whose composition in turn varies with the prevailing seasonal sea ice conditions.

## 1. Introduction

Underwater acoustic environments (also referred to as ‘soundscapes’) are a complex assembly of all of the sounds produced by marine species (biophony), environmental conditions (geophony) and anthropogenic activities (anthropophony), which represent spatiotemporally unique soundscape patterns. While soundscapes represent a vital information resource for communication, orientation and foraging for a variety of marine organisms [1–3], many species are increasingly being affected and disturbed by soundscape alterations, owing to changes in environmental conditions and anthropogenic noise [4–6].

In polar areas, sea ice, icebergs and wind have a primary role in shaping ambient sound levels (e.g. [7–10]). However, in the Arctic Ocean (AO) the Arctic amplification, i.e. the rate of warming of this region which is almost four times the global average [11], has led to substantial changes and reductions in sea ice cover [11,12], resulting in higher ambient sound levels (e.g. [7]). The observed elevated ambient sound levels are primarily attributed to increased wind-mediated sounds owing to larger open water areas and cryonic sounds associated with unstable sea ice cover [7]. In the Southern Ocean (SO) increasing climate instability may lead to additional increases of sounds generated by breaking icebergs and glacier calving affecting ambient sound levels (e.g. [9]). This highlights the importance of considering the complex relationships among wind patterns and sea ice dynamics on overall soundscape characteristics in polar regions. In Arctic seas, sea ice decline promotes anthropogenic activities, including commercial shipping, fishing and tourism, as well as oil and gas exploration, which further increase underwater sound levels [13,14].

While the AO basin might still be considered acoustically pristine [15] as long as the sea ice cover remains some Arctic seas currently experience strong anthropogenic noise disturbance [14,16,17]. One example in the high Arctic is the Fram Strait (FS), which connects the AO basin with the Greenland Sea and is already experiencing seasonally dominating airgun and shipping noise (e.g. [18–20]), as well as annually increasing seasonal shipping activities [21]. By contrast, shipping noise in the SO is mainly connected to service traffic to research stations, while most of the SO stays traffic-free [14]. Tourism and fishing activities increase seasonally but are mainly concentrated off the Western Antarctic Peninsula [22]. Seismic airgun operations (scientific purpose only) also take place in the SO, albeit less frequently than in many Arctic regions (e.g. [20,23]). Marine mammals have formed on of the major sound sources in the SO that have always governed soundscape characteristics (e.g. [10,20]), when ignoring the major historical losses of cetaceans owing to commercial whaling [24], which must also have massively altered the underwater acoustic scene of the SO. Overall, the soundscape south of the Antarctic Convergence can currently still be considered relatively intact and quasi-pristine. In this regard, the SO could serve as a baseline to understand how intact polar marine soundscapes function, how they are composed and what characterizes them.

Recommendations of metrics to assess and monitor the *status quo* of marine soundscapes have focused mainly on ecoacoustic intensity metrics such as assessing the sound pressure level (SPL) of ambient sound (e.g. [20]), particularly in predefined frequency bands, such as within one-third-octave level (TOL) bands (e.g. [25–27]). However, characterizing marine soundscapes based solely on sound levels provides a one-dimensional view of the acoustic environment of a given habitat (e.g. [28]). Ecoacoustic diversity metrics, on the other hand, have the potential to capture the overall acoustic structure of the environment, i.e. the variability in the total spectro-temporal intensity distribution and complexity created by the ensemble of acoustic signals and how this is perceived.

To date, there are more than 60 different ecoacoustic metrics (EAMs) available, which extract and aggregate amplitude, time- and/or frequency-related variables into single values that are representative of intensity variability and complexity [29–32]. Of these, ecoacoustic diversity metrics have primarily been applied to investigate changes in terrestrial biodiversity patterns of acoustically active avian communities [33]. However, these metrics have yielded mixed results owing to the metrics’ sensitivity to changes in the signal-to-noise ratio or acoustic masking (e.g. [30,34]). Their usefulness for assessing and quantifying biodiversity has therefore been under debate, in marine and terrestrial ecosystems (e.g. [30,33,35]). Nevertheless, for characterizing marine and terrestrial soundscapes, to discriminate between different habitat types and ecosystem status, and investigating temporal shifts in soundscape heterogeneity, uniformity and periodicity, the combination of various EAMs including ecoacoustic diversity metrics are increasingly applied and has proven valuable (e.g. [32,36–43]). In marine habitats, the application of combinations of EAMs has shown promising results to discriminate between various marine ecosystems, such as distinguishing healthy and degraded reefs [36], deep-sea versus coastal reefs [36–38], or polar pelagic and on-shelf habitats [39].

Here, we characterize soundscapes of the Arctic eastern FS as an example of an anthropogenically affected, and the Antarctic Weddell Sea (WS) as an example of a quasi-pristine polar soundscape by applying unsupervised machine learning and a combination of ecoacoustic intensity and diversity metrics on passive acoustic monitoring data collected between 2016 and 2018. We thereby evaluate the suitability of recommended intensity metrics [25,26] and of some of the most commonly applied diversity metrics (e.g. [29,31]) to describe and distinguish overall soundscape characteristics and to evaluate whether these metrics can serve as standards to monitor the acoustic states of polar offshore environments. We thereby hope to contribute towards the aim of the International Quiet Ocean Experiment (IQOE) and Global Ocean Observing System [44] regarding the development of standardized sets of metrics for holistic ocean soundscape monitoring.

## 2. Material and methods

### 2.1. Data acquisition

**Figure 1.**
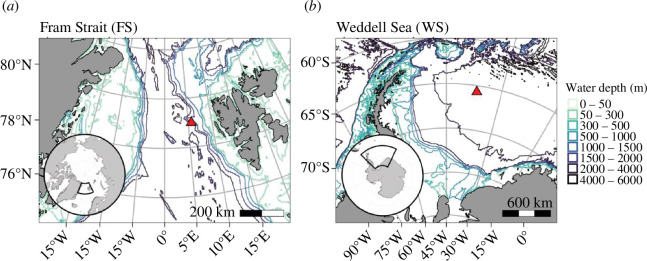
Geographic position of the recording stations. (*a*) FS, Arctic, recorder position 79 °N and 5.7 °E and (*b*) the WS, Antarctica, recorder position 65.7 °S and 36.7 °W. Maps were created with the R-package ggOceanMaps [45].

Acoustic data were collected in the eastern FS, AO, from 2016 to 2017 inclusive (herein referred to as FS station; figure 1) [46] and in the WS, Atlantic Sector of the SO, from 2017 to 2018 inclusive (herein referred to as WS station; figure 1) [47].

The FS recorder was attached to one of the oceanographic deep-sea moorings of the Frontiers in Arctic Marine Monitoring (FRAM) Ocean Observing System in the eastern FS [48]. This observatory is located in the pathway of the Atlantic Water inflow into the AO and West Spitsbergen Current [49]. The WS recorder formed part of a mooring within the Hybrid Antarctic Float Observation System (HAFOS) in the WS [50] within the Weddell Sea gyre [51]. Data for this study were selected to be offshore soundscape recordings from the eastern FS and the WS covering a similar period. The gain calibration differences of the hydrophones pre- and post-deployment were less than 1 dB for both recorders. Passive acoustic data were collected using autonomous acoustic Sono.Vault (Develogic GmBH, Hamburg) recorders with an omnidirectional hydrophone (RESON TC4037-3), set to sample continuously with a bit depth of 24 bits, storing the data in 10 min *.wav files (see table 1 for further details).

**Table 1.** Summary of the Sono.Vault recorder settings and deployment information.

region	deployment ID	position	recording period	months analysed	sampling rate (Hz)	recorder (water) depth (m)	pre- and post-calibration gain (dB)	sensor sensitivity (dB re 1 V µPa^−1^)
FS, AO	ARKF05-17 _SV1088	79 °N 5.7 °E	2016/07/23 –2017/07/18	August 2016 November 2016 February 2017 May 2017	48 000	808 (2100)	41.0/41.2	−193.1
WS, SO	AWI208-08 _SV1009	65.7 °S 36.7 °W	2017/01/19 –2018/05/02	May 2017 August 2017 November 2017 February 2018	6857	1032 (4766)	41.3/41	−192.5

The eastern FS station was selected to represent a polar soundscape strongly affected by anthropogenic noise and low to no sea ice cover, thereby providing a potential baseline for future scenarios of polar soundscapes in Arctic regions that are still considered pristine. The WS station was selected as a baseline representation of a quasi-pristine and intact soundscape containing a high bioacoustic diversity and experiencing seasonal sea ice cover. Based on a pre-screening of long-term spectral averages of both datasets using the open portal to underwater soundscapes (OPUS; CC-BY 4.0 AWI 2023; [52]), we selected four months of data per recording site (table 1) to represent the seasonal soundscapes in terms of the typical sound sources present over the course of 1 year.

### 2.2. Ecoacoustic metrics

In total, 14 EAMs were applied, of which nine are intensity metrics and five are diversity metrics, to characterize variability in ambient sound and spectro-temporal intensity distribution, respectively.

#### 2.2.1. Ecoacoustic intensity metrics

To compute the nine ecoacoustic intensity metrics (see electronic supplementary material, table S1 for an overview) in a standardized way, the MANTA (Making Ambient Noise Trends Accessible) software was used (v9.6.11 and v9.6.12, standalone version) [53]. MANTA computes the calibrated power spectral density (PSD; [54]) over 1-min time intervals (i.e. 10 data points per 10 min *.wav file) at a hybrid millidecade resolution [55,56]. The spectral probability density (SPD) [57,58] and the SPLs [59] were computed from the MANTA-derived PSD. The SPLs were computed for seven defined frequency bands: the broadband frequency range (10–3428 Hz, herein further referred to as broadband SPL) and six TOL bands, centred at 63, 125, 250 and 500 Hz, as recommended by the European Union Marine Strategy Framework Directive (MSFD; descriptor 11) [25] and Merchant *et al*. [26]. The sampling frequency of 6857 Hz was the smallest common denominator of the available frequency range for both datasets, which is why the SPL for the broadband frequency range was computed over 10–3428 Hz. Frequencies below 10 Hz were excluded to avoid a bias of low-frequency recorder-generated flow noise [60] on SPL measurements. The TOL bands were computed as decidecade bands as proposed by the IQOE [61]. To also describe the acoustic environment for species vocalizing at low frequencies (<50 Hz), such as fin whales (*Balaenoptera physalus*) and (Antarctic) blue whales (*B. musculus intermedia* and *B. musculus*), the 20 and 25 Hz TOL bands were included for the FS and WS stations, respectively.

The intensity metrics applied in our study are intended to characterize ambient sound [62], which is defined in our study as all sounds except for acoustic-self noise following the definition by the International Organization for Standardization (ISO) [63]. In this study, we apply the term ‘ambient sound’. We apply the term ‘noise’ when referring specifically to non-natural sounds, i.e. recorder-generated and anthropogenic signals.

#### 2.2.2. Ecoacoustic diversity metrics

The five ecoacoustic diversity metrics to describe soundscape characteristics, such as spectro-temporal heterogeneity or uniformity (see electronic supplementary material, table S2 for an overview), were computed using the functions provided in the R-packages, i.e. TuneR [64], Seewave [65] and Soundecology [66]. For direct comparability of the computed diversity metrics between the FS and the WS, the FS *.wav files were down-sampled (using the function ‘downsample’ of the package TuneR [64] in R) to a sampling rate of 6857 Hz, corresponding to the sample rate of the WS recordings. To avoid the aliasing of higher frequency signals on the one hand and to exclude frequencies below 10 Hz on the other hand, the audio data were band-pass filtered for 10–3428 Hz with a Finite Impulse Response filter (using the function ‘fir’ of the package seewave [65] in R, custom fitted to adhere to the original bit depth of 24) before down-sampling. All diversity metrics were computed for the broadband frequency range (10–3428 Hz) and for each 10 min *.wav file, resulting in one data point per 10-min file.

The Acoustic Complexity Index (ACI) measures intensity variation between two successive time bins and distinguishes high- from low-intensity variability, which are reflected as high and low ACI values, respectively [67]. The ACI has previously shown promise for distinguishing healthy from degraded reefs [36] and for data from the WS, for distinguishing on-shelf and pelagic soundscapes [39]. In our study, we consider increased ACI values to reflect greater temporal soundscape heterogeneity.

The Acoustic Evenness Index (AEI) and the Acoustic Diversity Index (ADI) indicate the degree of uniformity of the spectral intensity distribution by measuring intensity variation among frequency bins [68]. The AEI is based on the Gini coefficient, while the ADI is based on the Shannon’s Diversity Index [68]. Both indices measure the intensity saturation within each frequency band, which reflects the degree of spectral uniformity. The AEI responds conversely to acoustic patterns compared with the ADI, meaning that with increasing acoustic uniformity the ADI increases towards 1 and the AEI decreases towards 0.

The total acoustic entropy index (HI) computes the Shannon evenness of the amplitude envelope and estimates the spectral and temporal uniformity of the intensity distribution across acoustic space [69]. The HI ranges between 0, indicating a heterogenous intensity distribution and 1, indicating a uniform intensity distribution. This index has shown promise in distinguishing healthy from degraded reefs [36] and on-shelf and pelagic habitats in the WS [39].

The Bioacoustic Index (BI) is intended to describe the saturation of acoustic space of a defined temporal range, by measuring the area under the mean spectral curve for sound levels greater than the minimum sound level between two frequency limits [70,71]. The BI was developed to measure avian abundance, with an increase in BI corresponding to increasing call rates and the intensity of choruses [70,72]. Therefore, in the context of our study, an increase in the BI reflects an increase in spectral heterogeneity.

### 2.3. Post-processing

The WS mooring contained an oceanographic sound source emitting RAFOS (ranging and fixing of sound; [73]) signals daily at 12:39 UTC. These signals are upsweeps that lasted 80s, ranged from 259 to 261 Hz and had a source level of 175 dB re 1 µPa. To prevent biases in the metrics’ trends, the respective data points including the RAFOS signal were removed. For the ecoacoustic intensity metrics, the removal corresponded to four data points (each corresponding to a 1-min window) from 12:39 to 12:42 UTC. For the ecoacoustic diversity metrics, two data points (corresponding to two 10-min files) starting in the period of 12:28−12:42 UTC were discarded.

### 2.4. Environmental parameters

To interpret soundscape characteristics in the light of local environmental parameters known to affect polar soundscapes (e.g. [7,10]), data on the sea ice concentration (SIC) and wind speed were analysed for the recording positions. To spatially average the SIC and the wind speed across a defined area, the function ‘extract’ from the package raster [74] in R [75] was used, which averages across all pixels within the defined area boundaries.

#### 2.4.1. Sea ice concentration

The daily SIC was obtained from the University of Bremen [76] at a grid resolution of 3.125 × 3.125 km on a polar stereographic grid for both the AO and SO. SICs of ≤15% were considered to indicate ‘open water’ conditions. The daily SIC was averaged for radii of 30, 50 and 100 km around the recording site. The size of the areas over which the daily SIC was averaged covers the assumed propagation range of high-, mid- and low-frequency signals of most pinnipeds and cetaceans (e.g. [77–79]).

#### 2.4.2. Wind speed

The hourly east- and westward field components of wind speed 10 m above the Earth’s surface were obtained from the European Centre for Medium-Range Weather Forecasts from the ERA5 dataset for re-analysis [80]. The hourly wind speed was spatially averaged across a radius of 0.25° latitude (corresponding to 27.75 km) around the recording sites.

### 2.5. Statistical analysis

To explore the influence of wind speed and SIC on the soundscape characteristics we fitted multiple regression models, assuming linearity (e.g. [7,10,81]) and fitting wind speed, SIC and the season as predictor variables and the hourly mean of ten EAMs (the broadband SPL, 63, 125, 250 and 500 Hz TOL band, ACI, AEI, ADI, HI and BI) as response variables. As the 20 and 25 Hz TOL bands were only measured for either the FS or the WS, and the 20 Hz band was strongly affected by flow noise, these TOL bands were excluded from further analysis. We included an interaction between wind speed and SIC to model the effect of sound generated by unstable sea ice moved by wind (e.g. [7]) and the effect of stable sea ice cover dampening the influence of wind on ambient sound levels (e.g. [10]). We differentiated between seasons to account for seasonal variation in the vocal activity of marine mammals and/or anthropogenic noise, which could cause deviation from the linear response of sea ice and wind speed on the metrics. For the broadband SPL, the 63 and 125 Hz TOL bands measured at the FS station a generalized linear gamma regression model using a log-link was applied. A general linear regression model was applied for the 250 and 500 Hz TOL band with a log link and an identity link, respectively, for the FS station. General linear models were fitted for the broadband SPL, 63, 250 and 500 Hz TOL band with an identity link and for the 125 Hz with a log-link at the WS station. A β-regression model, applying the ‘betareg’ function from the R package betareg [82] was applied for the AEI and the HI, as both metrics are bounded between 0 and 1. For the AEI, a log-link and for the HI a logit-link was applied. A generalized linear gamma regression with a log-link was fitted for the ACI and ADI for both regions, and for the BI for the FS only. In addition, the ADI of the WS was inverse transformed to follow a gamma distribution. A general linear model was fitted for the BI in the WS. General and generalized linear models were implemented using the ‘glm’ function in R [75].

To explore differences and similarities between the seasonal soundscape characteristics of the FS station and the WS station, we applied k-means clustering. The hourly mean of five intensity metrics, the broadband SPL, 63, 125, 250 and 500 Hz TOL band, and the hourly mean of the five-diversity metrics ACI, AEI, ADI, HI and BI, resulting in 5756 soundscape observations were used. As the 20 and 25 Hz TOL bands were only measured for either the FS or the WS, and the 20 Hz band was strongly affected by flow noise, these TOL bands were excluded. Before applying the k-means clustering, we tested for the cluster tendency of our dataset by applying Hopkins Statistics [83] using the function ‘get_clust_tendency’ from the R package factoextra [84], which yielded a significant cluster tendency (H > 0.9). The data were then standardized by scaling to zero mean and unit variance by applying the ‘decostand’ function from the package vegan in R [85]. Following the approach by Roca and Van Opzeeland [39], we used the function ‘cascadeKM’ from the vegan package in R [85] to identify the number of clusters. We tested for 2–8 clusters (since we had two sites with four seasons each) and used the Simple Structure Index (SSI) [86] to determine the best number of clusters, as indicated by the highest SSI value. The SSI combines the maximum difference of each variable to a cluster prototype, the difference between the mean of variable values in each cluster prototype, and the overall mean of variable values for all clusters, which influences the cluster solution [86]. We then applied the k-means clustering algorithm on the hourly mean of the ten EAMs, applying the R built-in function ‘kmeans’ [75]. Furthermore, a principal component analysis (PCA) for feature selection using R’s built-in function ‘prccomp’ [75] was applied to investigate that EAMs had the greatest explanatory power (contribution >10%) in explaining the variance of the clustered soundscape characteristics. To visualize the soundscape variation of the cluster analysis, we used a PCA biplot. All of the statistical analyses were conducted in R (v4.3.0) [75].

## 3. Results

### 3.1. Dominant sound sources

**Figure 2.**
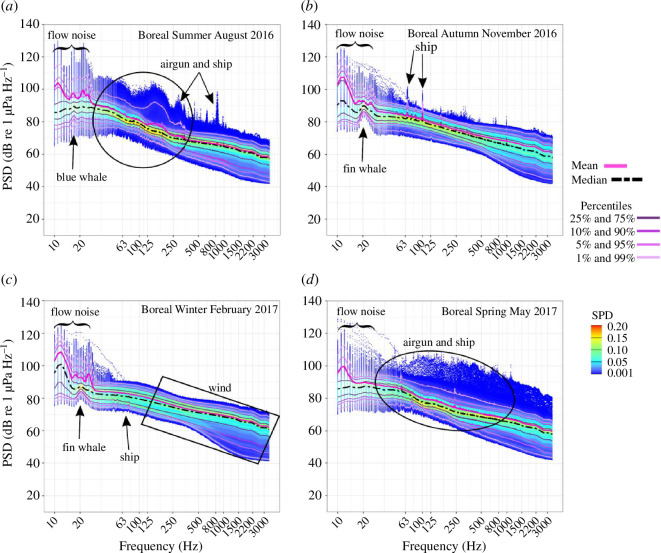
(*a*–*d*) Seasonal mean (pink line) and median (black-dashed line) power spectral density levels (PSD, dB re 1 µPa Hz^−1^) and spectral probability density (SPD, colour scale) of the FS recordings. The main contributors to seasonal soundscape patterns are indicated by black arrows, boxes and circles. The indicated wind noise in *c* is exemplary and also applies to the other panels.

At the FS station, seasonal variation in the PSD and SPD was relatively low and could be attributed to the dominance of anthropogenic noise from seasonal (boreal summer, autumn and spring) airgun operations, as well as year-round ship noise and wind-mediated sounds (figure 2). However, airgun and ship noise were most pronounced in the PSD and SPD in the boreal summer and spring (figure 2*a,d*) and intense airgun and ship signals caused a high scattering of the SPD (figure 2*d*). In the boreal autumn, airgun noise was present only in the first week and had little effect on the seasonal PSD and SPD. Wind-mediated sounds increased the scattering of the SPD particularly in the higher frequencies (figure 2). The influence of biophonic sound sources (marine mammals) on the mean PSD was minimal (figure 2). The peaks in the lower percentiles (1–25%) and median PSD at 18 Hz and 20 Hz, from boreal summer to winter, indicated the acoustic presence of blue and fin whales, respectively (figure 2*a–c*). The peaks in the mean PSD from 12 to 25 Hz across all seasons were identified as flow noise (figure 2) and thus represent an acoustic artefact at the recorder and not a true component of the soundscape.

**Figure 3.**
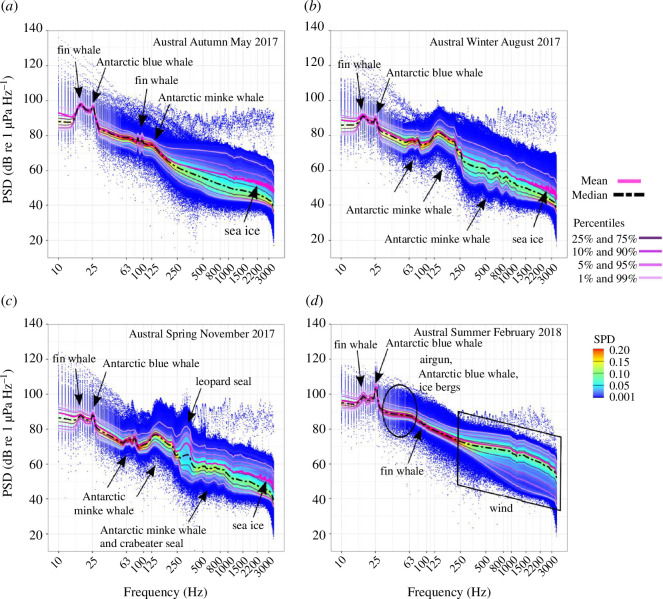
(*a*–*d*) Seasonal mean (pink line) and median (black-dashed line) power spectral density levels (PSD, dB re 1 µPa Hz^−1^) and spectral probability density (SPD, colour scale) of the WS recordings. The main contributors to seasonal soundscape patterns are indicated by black arrows, boxes and circles.

At the WS station, the year-round dominant sound sources were of natural origin (marine mammals, wind-mediated, sea ice and icebergs). The presence of anthropogenic sound sources was minimal, with seismic survey airgun pulses being detected only on a few days in the austral summer. Distinct peaks in the mean PSD curve revealed pronounced seasonal variation governed by the acoustic presence of five marine mammal species (figure 3): fin whales (20 Hz, across all months, as well as 86 and 99 Hz in the austral autumn and summer), Antarctic blue whales (28 Hz, across all months), Antarctic minke whales (*B. bonaerensis*; 60–1000 Hz in the austral autumn to spring), leopard seals (*Hydrurga leptonyx*; 300 Hz in the austral spring), and crabeater seals (*Lobodon carcinophaga*; 350–1000 Hz in the austral spring). The broader scattering of the SPD at frequencies above 250 Hz was likely a consequence of broadband sea ice and wind-mediated sounds (figure 3). In addition, in the austral summer, wind-mediated sounds increased the PSD in the absence of sea ice cover, and the plateau of the mean and median PSD in the range of 25–70 Hz likely reflected the interplay of airgun operations, Antarctic blue whale D-calls and supposedly iceberg sounds (figure 3*d*).

### 3.2. Ambient sound levels

**Figure 4.**
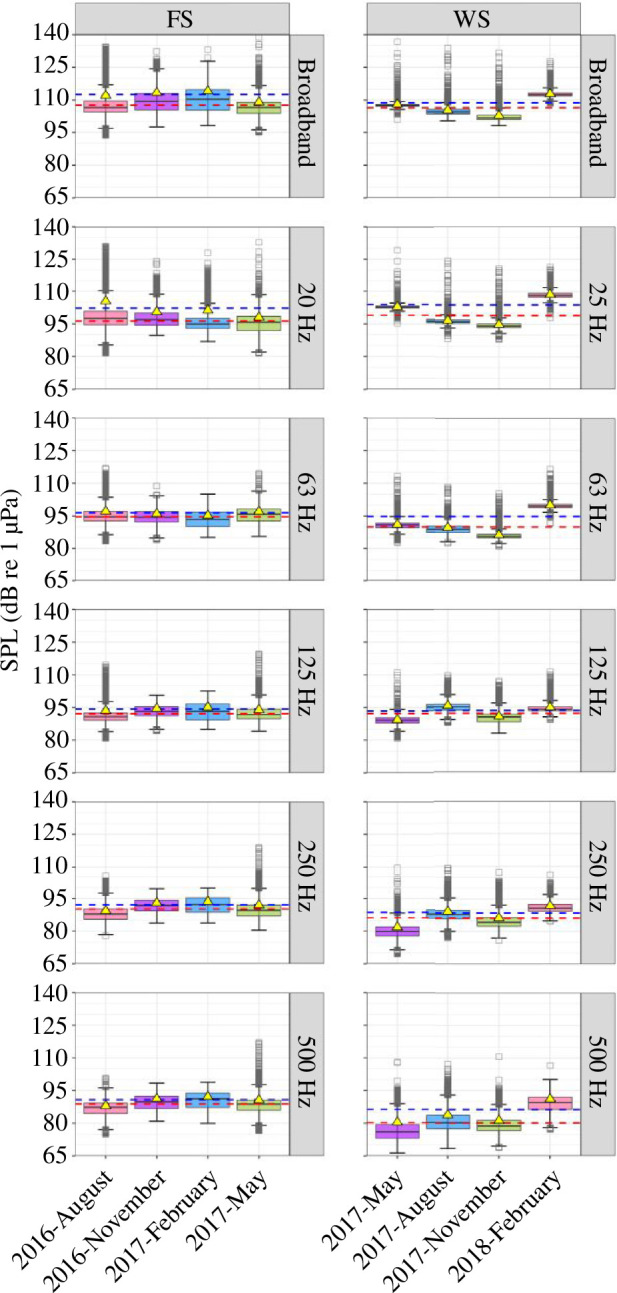
Ecoacoustic intensity metrics as SPLs for the broadband (10–3428 Hz) and TOL bands centred at 20 Hz (for the FS station), 25 Hz (for the WS station), and 63, 125, 250 and 500 Hz. The yellow triangles indicate the seasonal mean SPLs and the blue- and red-dashed lines indicate the annual mean and median SPLs, respectively. The box colours indicate the seasons: pink for summer, purple for autumn, blue for winter and green for spring. The lower and upper box borders show the interquartile range (25th to 75th percentiles) and the whiskers indicate the 5th and 95th percentiles. Seasonal medians are given by the inner black lines (see also electronic supplementary material, tables S3 and S4, for mean, median, maximum and minimum values for the FS station and the WS station, respectively).

At the FS station, the seasonal variability of the mean SPLs for all frequency bands was low, but the intra-seasonal variability of SPLs was high as indicated by the width of the interquartile range (figure 4, electronic supplementary material, table S3). Conversely, at the WS station, the mean SPLs for all frequency bands showed seasonality, while intra-seasonal variability of SPLs was generally low but increased for the 500 Hz TOL band (figure 4, electronic supplementary material, table S4).

The FS station exhibited higher annual median and mean SPLs for all of the investigated frequency bands compared with the WS station, with values of 107.6 and 112.5 dB re 1 µPa at the FS station, and 106.4 and 108.7 dB re 1 µPa at the WS station (figure 4), respectively. The median SPL measured in the austral summer at the WS station exceeded all of the seasonal median SPLs measured at the FS station in the 63 Hz TOL band. The lowest single broadband SPL was measured in the boreal summer at the FS station (93.8 dB re 1 µPa) and in the austral spring at the WS station (98.3 dB re 1 µPa). The highest single broadband SPLs were measured in the boreal spring in the FS (138.7 dB re 1 µPa) and in the austral summer at the WS station (142.2 dB re 1 µPa). At the FS station, the highest median SPLs for the 63 Hz TOL band were measured in the boreal spring (95.8 dB re 1 µPa) for the 125 Hz TOL band in the boreal autumn and winter (both, 93.3 dB re 1 µPa), and for the 250 and 500 Hz TOL band in the boreal winter (92.3 and 91.3 dB re 1 µPa, respectively). At the WS station, the highest median SPLs for the 63, 250 and 500 Hz TOL bands were measured in the austral summer (99.6, 90.7 and 89.6 dB re 1 µPa, respectively) and in the austral autumn for the 125 Hz TOL band (95.2 dB re 1 µPa).

At the FS station, the 20 Hz TOL band, which was applied to describe ambient sound levels within the communication range of fin and blue whales, was highly affected by flow noise (figure 2) and was therefore not interpretable. The 63 and 125 Hz TOL bands demonstrated sensitivity to airgun noise and the 250 Hz band was indicative of ship noise.

For the WS, the 25 Hz TOL band varied with the seasonal intensity variation of fin and Antarctic blue whale choruses (figure 4). In the austral summer in the WS, the 63 Hz TOL band was affected by Antarctic blue whale D-calls, airgun operations, and broadband bursts by supposedly distant iceberg sounds in austral summer. The 125 Hz band was also sensitive to the presence of airgun noise in the austral summer. The 63, 250, 125 and 500 Hz TOL bands were sensitive to intensity fluctuations in Antarctic minke whale choruses from the austral autumn to spring. The 250 and 500 Hz bands reflected variation in the intensity of leopard and crabeater seal choruses during the austral spring.

The 500 Hz band best reflected temporal patterns in wind speed in both regions.

### 3.3. Spectro-temporal intensity variability

**Figure 5.**
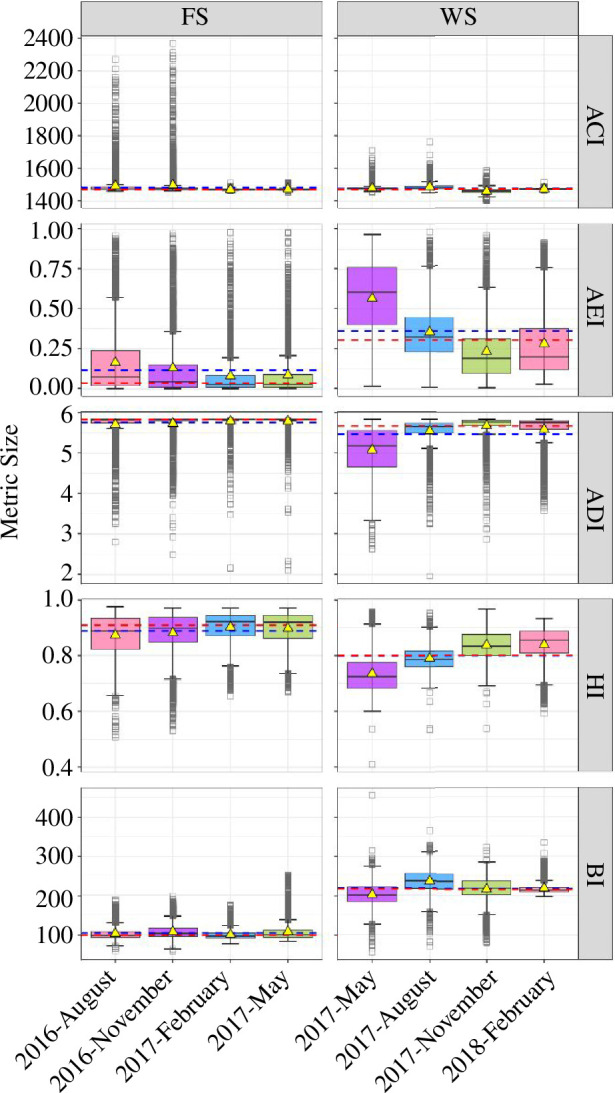
Ecoacoustic diversity metrics (ACI, AEI, ADI, HI and BI) for the FS and WS. The yellow triangles indicate the seasonal metric means and the blue- and red-dashed lines indicate the annual metric means and medians, respectively. The box colours indicate the seasons: pink for summer, purple for autumn, blue for winter and green for spring. The lower and upper box borders show the interquartile range (25th to 75th percentiles) and the whiskers indicate the 5th and 95th percentiles. Seasonal medians are given by the inner black lines.

Overall, the ACI showed high temporal uniformity at the FS station and at the WS station across seasons but increased in response to repetitive impulsive broadband sounds such as clicks, pulses, cracking and squeaking or rubbing sounds. At the FS station, ACI peaks were related to the high-intensity click sequences of sperm whales (*Physeter macrocephalus*; boreal summer and autumn; see outliers in figure 5). At the WS station, the ACI responded to the chirps of Weddell seals (*Leptonychotes weddellii*; austral autumn), Antarctic minke whale pulses (in the austral autumn, winter and spring), and the cracking sounds of sea ice (in the austral autumn to spring; see outliers in figure 5).

Spectral heterogeneity (AEI) and uniformity (ADI) did not show pronounced seasonality at the FS station (figure 5). Here, both diversity metrics also had relatively low (AEI) and high (ADI) seasonal means (figure 5) reflecting the presence of continuous broadband wind-mediated sounds, ship and airgun noise. The AEI increased and the ADI decreased strongly in the presence of flow noise when wind-mediated sounds were absent. By contrast, in the WS, the AEI and the ADI showed a pronounced seasonal pattern (figure 5). The AEI decreased and ADI increased towards the austral spring as various marine mammal choruses saturated several frequency bands at the same time (figure 5). In the austral summer, the broadband wind-mediated sounds and sounds of icebergs caused a decrease in the AEI and an increase in the ADI.

At the FS station, broadband wind-mediated sounds, ship and airgun noise caused year-round high HI values (figure 5). Drops in the HI were mainly caused by the absence of broadband wind-mediated sounds, while high-intensity sounds, e.g. from flow noise or a continuous fin whale chorus, were concentrated in the lower frequencies (i.e. 10–30 Hz). In the WS, marine mammal choruses simultaneously saturated multiple frequency bands resulting in high HI values.

The seasonal means of the BI were consistently low at the FS station (figure 5), which we attribute to broadband wind-mediated sounds, ship and airgun noise across seasons causing constant spectral uniformity. At the WS station, higher mean BI values indicated higher spectral heterogeneity and the observed seasonal variability (figure 5) was caused by variation in the spectral heterogeneity, attributed to the characteristics and intensity of the marine mammal choruses and wind-mediated sounds.

### 3.4. Environmental effects on the acoustic metric response

At both polar recording sites, no significant differences were found between the SIC around the recording site for the three investigated radii (30, 50 and 100 km), and therefore, only the SIC for the 50 km radius was further analysed.

At the FS station, sea ice was absent throughout the year (with the minor exception of two weeks at the end of boreal spring with SICs of 15–25%; electronic supplementary material, figure S1), while the WS station experienced open water conditions only in the austral summer but indicated a rather closed cover with SICs >79.7% in all of the other austral seasons (electronic supplementary material, figure S2).

At the FS station, the regression models indicate that increasing wind speed and the absence of sea ice cover influenced the SPLs of all frequency bands positively, with the greatest variation among seasons being found for the 500 Hz TOL band (electronic supplementary material, table S5 and figure S1). The low effect sizes (*R*² ≤ 0.3, electronic supplementary material, table S5) for the broadband SPL, 63 and 125 Hz TOL band likely indicate a strong influence of anthropogenic noise in these bands, while the self-noise likely caused the low effect size (*R*² = 0.153) in the broadband SPL. By contrast to the SIC the wind speed affected the diversity metrics, but in different ways for each metric, and the effect sizes were generally low (*R*² < 0.5, electronic supplementary material, table S6). Moreover, seasonal variation was negligible for the diversity metrics at the FS station (electronic supplementary material, table S6 and figure S3). The ACI, AEI and BI were negatively, while the ADI and HI were positively associated with wind speed. Only the AEI, HI and BI were affected by the interaction of wind speed and sea ice, owing to the small increase of SIC in boreal spring (electronic supplementary material, table S6 and figure S3).

At the WS station, the SPL of the 250 and 500 Hz TOL band were strongly influenced by the wind speed during absent sea ice cover in austral summer while sea ice cover dampened the influence of wind speed in the other seasons (electronic supplementary material, table S5 and figure S2). The low influence of wind speed in the remaining frequency bands is likely owing to interference with lower frequency (<200 Hz) marine mammal vocalizations and sound from breaking icebergs or calving glaciers. Interestingly, the SIC alone did not have a significant effect on the ACI but the interaction with wind speed did (electronic supplementary material, table S6 and figure S4). For the other metrics (AEI, ADI, HI and BI) high SIC dampened the effect of the wind speed on the metrics. Furthermore, the SIC in austral spring seemed to have only a weak or inverse influence on these metrics compared to the other sea ice-covered austral seasons (autumn and winter, electronic supplementary material, table S6 and figure S4).

### 3.5. Statistical comparison of soundscapes

**Figure 6.**
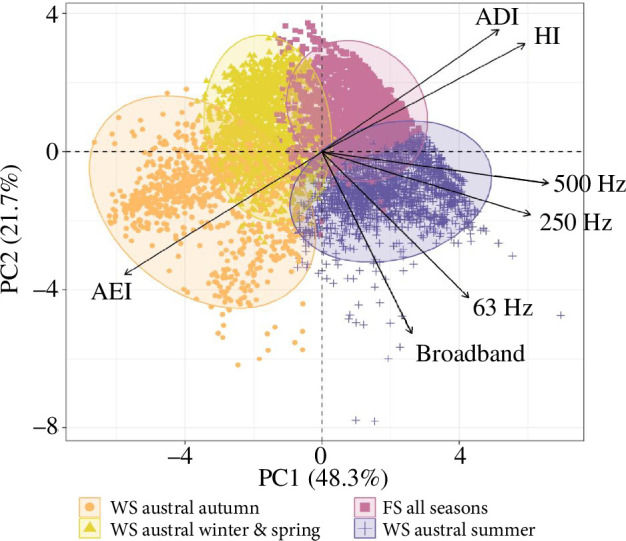
The PCA biplot for feature selection shows the clustered soundscape characteristics (table 2) explained by the combination of the hourly means (5756 soundscape observations) of the ten EAMs (broadband SPL, 63, 125, 250 and 500 Hz TOL band, ACI, AEI, ADI, HI and BI) along the first two principal components (PCs). Ellipses represent the 95% percentiles of cluster variables. The length of the arrows indicates the importance of the seven EAMs, which together explain approximately 70% of the variance in the first two PCs in differentiating among soundscape characteristics.

The PCA for feature selection revealed that the first two principal components (PCs) explained 70% of the total variance in the soundscape characteristics (figure 6 and electronic supplementary material, table S7). The variance among the soundscape characteristics in the first two PCs was best explained by the AEI, HI, 500 Hz, 250 Hz, ADI, 63 Hz and broadband SPL (figure 6 and electronic supplementary material, figure S5). The 125 Hz, BI and ACI were deemed less important in explaining variance in the first two PCs. Moreover, k-means clustering using the SSI criteria identified four clusters that best explained the variation across regions and seasons in soundscape characteristics (figure 6 and table 2).

**Table 2.** Soundscape characteristics contributing to cluster formation. Clusters were obtained by applying k-means clustering on the hourly means of ten EAMs (broadband SPL, 63, 125, 250 and 500 Hz TOL band, ACI, AEI, ADI, HI and BI). The clusters are named according to their greatest contributors.

clusters		FS	WS		
		all seasons	austral autumn	austral winter and spring	austral summer
number of total soundscape observations		1731	765	1558	1702
FS	August 2016 (boreal summer)	30.2%	5.5%	2.2%	8.5%
	November 2016 (boreal autumn)	19.2%	5.9%	0.4%	19.7%
	February 2017 (boreal winter)	15.7%	2.2%	1.4%	21.2%
	May 2017 (boreal spring)	27.7%	2.9%	1.4%	13%
WS	May 2017 (austral autumn)	2.5%	66.1%	12.3%	0.1%
	August 2017 (austral winter)	0.2%	3.7%	41.7%	3.6%
	November 2017 (austral spring)	4.5%	1.7%	40.3%	0.1%
	February 2018 (austral summer)	0%	12%	0.2%	33.8%

The soundscape observations of all boreal seasons of the FS were summarized within one cluster exhibiting high spectral uniformity caused by broadband wind-mediated sounds, airgun and ship noise. We consider the similarities in soundscape observations found between the FS and the WS in the cluster comprising mostly austral summer observations (table 2), to be an artefact explained by the low-frequency flow noise at the FS station causing a higher spectral heterogeneity in the data. Moreover, the k-means clustering did not seem to effectively distinguish anthropogenically affected periods in the austral summer in the WS, or variation in anthropogenic noise occurrence at the FS station (e.g. airgun noise versus ship noise in the boreal winter).

Seasonal differences in soundscape characteristics at the WS station are reflected by variation in three clusters separating the austral autumn, the austral summer and the combined austral winter and spring (table 2, figure 6). The cluster comprising mainly soundscape observations of the austral autumn reflects characteristics with spectral heterogeneity. These are governed by the absence of wind-mediated sounds due to sea ice cover, and variability in spectral intensity owing to the marine mammal choruses. The austral winter and spring cluster reflects characteristics with higher spectral uniformity and low sound levels. The similarity between these seasons is attributed to the absence of wind-mediated sounds linked to sea ice cover and the presence of a rich and diverse acoustic marine mammal community with relatively consistent chorus intensity. The austral summer cluster reflects soundscape characteristics with elevated sound levels governed by broadband wind-mediated sounds and intense vocalizations by fin whales and Antarctic blue whales in lower frequencies, additionally causing a lower spectral uniformity compared with the FS cluster.

## 4. Discussion

We applied a set of 14 ecoacoustic intensity and diversity metrics to characterize seasonal soundscapes of one recording site in the Arctic FS and one recording site in the Antarctic WS to investigate broad differences between anthropogenically affected and pristine polar offshore soundscape characteristics. We furthermore aimed to produce a baseline for understanding intact polar soundscapes. Moreover, we tested the suitability of a set of nine intensity and five diversity metrics in this context, and were able to show that this set of EAMs seems adequate to capture overall spatiotemporal differences in polar offshore soundscape characteristics and acoustic states. Our results suggest that soundscape characterization using a multi-metric approach including intensity and diversity metrics provides a valuable basis for assessing the acoustic *status quo* of remote polar offshore environments.

### 4.1. Differences between anthropogenically affected and pristine polar offshore soundscapes

One of our key findings with respect to how both regions differ acoustically was the pronounced seasonality of the soundscape characteristics of the WS station compared with the sea ice-free and anthropogenically affected FS station. Overall, the soundscape of the FS station exhibited relatively constant ambient sound levels and high spectro-temporal uniformity year-round governed by constant wind-mediated sounds and anthropogenic noise. The soundscape of the WS station exhibited pronounced seasonal soundscape characteristics with a higher degree of spectral heterogeneity and lower intra-seasonal variability in ambient sound levels compared with the station in the FS. The pronounced seasonal pattern was governed by the seasonal vocal activity of the marine mammal community composition causing varying saturation levels of the acoustic space linked to the seasonal sea ice patterns.

#### 4.1.1. Overall ambient sound levels

At the FS station, the seasonal median SPLs for the 63–500 Hz TOL bands closely aligned with the annual median SPLs for the same TOL bands in the sea ice-covered western FS ranging from 83.9 to 94.6 dB re 1 µPa between 2008 to 2013 [18]. Similar to the western FS [18], we also found an effect of aigun pulses and distant fin whale calls on the PSD. Although we found no influence of bowhead whale (*B. mysticetus*) calls on the PSD at the eastern FS station compared with the western FS [18], a weak influence by blue whale calls in boreal summer was found to be similar to the observation of Klinck *et al*. [19]. At the WS station, the median SPLs for the 63–500 Hz TOL bands ranged from 80.2 to 92.2 dB re 1 µPa. These values correspond to the range of values reported by Dziak *et al*. [87] for the Bransfield Strait, where sound levels from 70 to 90 dB re 1 Pa/Hz were documented for 51–90 Hz. The intra-seasonal variability within the measured frequency bands for each region in our study was also consistent with the observation of Haver *et al*. [20] that the variability of ambient sound levels at the FS station is higher owing to inconsistent changes in anthropogenic noise overlapping with other sound sources. By contrast, lower variability in ambient sound levels in the SO was caused by seasonally consistent marine mammal calling and weather patterns [20]. The annual median broadband SPL was similar between the two regions and was in the same range as the observations of Haver *et al*. [20], for the FS and the Bransfield Strait, SO, during 2009 and 2010. Together with our results, this suggests that the overall ambient sound levels in these two regions have not changed considerably over the last decade. For the FS, this seems surprising considering the increase in ships at Spitsbergen [21] and that global anthropogenic noise emissions are assumed to have doubled over the last decade [14] and increased even more quickly in shallow (sub-)Arctic areas (i.e. Norwegian, Barents and Kara Sea) [14]. However, with the opening of the Arctic likely leading to the establishment of the Trans-Polar Sea Route [21], ambient sound levels in the eastern FS will likely increase in the future.

The National Oceanic and Atmospheric Administration National Marine Fisheries Service [88] and Southall *et al*. [89] define a threshold of 120 dB re 1 µPa for continuous anthropogenic noise with the potential to harass marine mammals. Moreover, Halliday *et al*. [13] mention that wind-mediated sounds alone can increase ambient sound levels above 120 dB re 1µPa in the Arctic. At the FS station, ambient sound levels within the TOL bands of and over 63 Hz did not appear to surpass this threshold. However, even if this threshold is not reached or exceeded, this does not imply that the anthropogenic noise is not harming or disturbing for marine mammals in this region. At the WS station, occasional single SPL measurements, potentially stemming from earthquakes and icebergs [8,9,90], exceeded 120 dB re 1 µPa. The source level of fin and Antarctic blue whale vocalizations can reach 189 dB re 1 µPa @ 1 m [77] and therefore easily exceed this threshold when produced nearby. This means that anthropogenic noise-affected soundscapes are not necessarily characterized by high-amplitude ambient sound levels. Consequently, the intensity of natural, i.e. biophonic and geophonic, sounds should not be underestimated as a cause of high sound levels, and the interpretation of ambient sound levels requires sound source context.

#### 4.1.2. Environmental sounds

The soundscape characteristics of both regions were significantly influenced by wind-mediated sounds but to different extents. Owing to a lack of year-round sea ice cover at the FS station, wind-mediated sounds caused increased and intra-seasonal variation in ambient sound levels as well as spectro-temporal uniformity year-round. At the WS station, soundscape characteristics were governed by the seasonal sea ice cover from the austral autumn to spring, and hence wind-mediated sounds only affected soundscape characteristics strongly in the sea ice-free austral summer. In addition, the sounds caused by breaking and collapsing icebergs increased ambient sound levels and spectral uniformity in the austral summer. In the austral winter, stable sea ice cover dampened wind-mediated sounds, while marine mammal vocalizations defined seasonal soundscape characteristics, consistent with the observation of Menze *et al*. [10]. However, corresponding to observations in sea ice-covered regions of the AO [7,18], strong winds during the freezing and melting seasons created cryonic sounds in the WS. These impulsive cracking or broadband abrasive sounds and harmonic tremors increased ambient sound levels, temporal heterogeneity (ACI) and spectral uniformity (AEI, ADI, HI and BI) at the WS station.

With ongoing climate change, sea ice regimes in the SO including the WS are expected to shift [91,92]. Annual ambient sound levels are therefore not only expected to rise in the AO but also in the SO as a consequence of unstable sea ice conditions, increased iceberg volume and extended periods of open water [7,9]. The increasing instability of the sea ice cover and the prolongation of open water periods might, furthermore, also change patterns of spectro-temporal uniformity owing to increasing effects of wind speed on soundscape characteristics.

#### 4.1.3. Marine mammal acoustic presence

At the FS station, biophonic richness varied seasonally and was highest in the boreal summer and autumn, with blue and sperm whales and fin and sperm whales, respectively, being acoustically present (figure 2). In the boreal winter, only fin whales were detected (figure 2). This seasonal pattern of these species’ acoustic presence corresponds to the results of Klinck *et al*. [19] from 2009 to 2010 and Ahonen *et al*. [93] from 2008 to 2018 in the FS, respectively. This consistency of acoustic presence patterns over the past decade supports previous observations (e.g. [93–95]) suggesting that the eastern FS provides a consistently seasonally suitable habitat for these species, likely as a result of the borealization of the AO (e.g. [93–96]). Linked to climate change and the borealization of the FS is the increasing period of open water and prey availability for seasonally migrating whales, including fin and blue whales, which has likely resulted in the observation of distributional changes north of 80°N and increases in the local abundance of these species [93–96]. Of the Arctic endemic marine mammals known to occur in the FS and around Spitsbergen (bearded seals (*Erignathus barbatus*), belugas (*Delphinapterus leucas*), narwhals (*Monodon monoceros*), and bowhead whales) [94,95,97] only bowhead whale vocalizations were acoustically identified with certainty at the FS station [98]. However, these vocalizations were not prominent at this recorder position [98] and hence unlikely to have significantly affected the overall soundscape characteristics.

In contrast to the FS station, biophonic richness at the WS station was highest during the austral winter. The WS station exhibited an overall richer biophony, with six marine mammal species contributing to seasonal soundscape characteristics (figure 3): two associated with open water conditions, i.e. fin and Antarctic blue whales, and four pagophilic species, i.e. Antarctic minke whales, leopard, crabeater and Weddell seals. Overall, their seasonal acoustic presence corresponds with the results of other PAM studies in the WS [10,99,100]. This suggests a basin-wide acoustic distribution of these species, and thereby, further highlights the year-round importance of the WS basin as a habitat for various marine mammals. All of these species, except for the sparse acoustic presence of Weddell seals detected by an increase in the ACI, affected and shaped the overall ambient sound levels and spectro-temporal uniformity.

Marine mammal choruses, had the strongest effect on the seasonal soundscape characteristics at the WS station, while the impact was weak at the FS station. In the WS, the year-round fin and Antarctic blue whale choruses with seasonal intensity variability are distinct soundscape characteristics, as also observed in other regions of the SO (e.g. [10,101]). The high intensity of these choruses is likely caused by higher species abundance or more localized presence at the WS recording site [101–103]. In the sea ice-covered seasons, the co-occurrence of intense choruses of Antarctic minke whales, leopard and crabeater seals in addition to the weaker but still prominent chorus of fin and Antarctic blue whales, saturated the temporal and spectral acoustic space. This caused a lower spectral heterogeneity (AEI, ADI and HI) over the course of the sea ice-covered seasons and aligns with previous terrestrial-based studies investigating the effect of high biophonic diversity and species richness on spectral uniformity [68,104]. Nevertheless, the rich biophonic diversity still maintains a higher degree of spectro-temporal heterogeneity in comparison to, for example, the presence of broadband and consistent wind-mediated sounds and additional anthropogenic noise such as at the FS station. This higher heterogeneity of co-occurring choruses can potentially be attributed to the hypothesis of species’ unique acoustic spectro-temporal niche occupancy to avoid overlap of vocalizations [105]. In addition, the seasonal variability of ambient sound levels and spectro-temporal heterogeneity observed at the WS station, which is caused by the seasonal acoustic niche separation of these species, stands out as a key feature for an intact polar offshore soundscape.

In contrast, the FS station did not exhibit this chorus-driven effect on soundscape characteristics as blue and fin whale choruses were weak (figure 2) and the population size of both species is estimated to be low in the FS [94,95]. Furthermore, a potentially greater distance to the recorder may have limited the influence of fin and blue whales on the FS soundscape characteristics. Moreover, the sensitivity of the diversity metrics to changes in the signal-to-noise ratio, arising from wind-mediated sounds (e.g. [30,81]), the masking effect of anthropogenic noise (e.g. [34]), and the flow noise in the FS recordings, may have affected the influence of fin and blue whale choruses on the soundscape characteristics.

Distributional and compositional shifts in acoustic marine mammal communities owing to changes in sea ice regimes and prey availability are not only expected for the FS (e.g. [93,95,96]) but also in the WS [99,100]. Such sea ice-linked geographic shifts and changes in abundance may result in major modifications in the overall soundscape characteristics governed by changes in acoustic species composition and chorus intensity. In the AO, increases in fin and blue whale abundance might locally also result in more pronounced choruses, potentially increasing soundscape variability. Conversely, in the SO, reductions in sea ice habitats may potentially lead to geographic shifts in pagophilic marine mammals, which might result in a local weakening or loss of seasonal variability in soundscape characteristics as their choruses become locally fainter or are lost.

#### 4.1.4. Anthropogenic noise

Our results imply that anthropogenic noise is present year-round in the eastern FS. The seasonal occurrence of airgun and ship noise aligns with the observations of Klinck *et al.* [19] and Haver *et al.* [20] for the same region. Moreover, airgun pulses probably contributed to intra-seasonal variation in ambient sound levels, which is also assumed by Haver *et al.* [20]. However, the merging of airgun noise also increased temporal uniformity, similar to the marine mammal choruses in the WS. Furthermore, anthropogenic noise contributed to a more stable spectral uniformity in the FS. This contrasts the findings of Wilford *et al.* [40], which were indicative of low uniformity for soundscapes containing airgun pulses. However, these pulses did not merge into a broad and continuous band such as the one observed at the FS station.

By contrast, the WS remains largely free from anthropogenic noise. Additionally, human activities in the SO are mostly limited to sea ice-free areas and seasons [20,22,23]. In this study, airgun pulses were also identified at the WS station but these were limited to a few days in the austral summer and did not seem to affect the soundscape characteristics of the WS. This might be explained by the similar impulsive characteristics of iceberg sounds and generally higher ambient noise levels attributed to wind-mediated sounds in the austral summer.

However, without appropriate adaptations of regulations governing human activities in polar oceans, further sea ice loss and resulting changes in ecosystem functions may lead to increasing anthropogenic activities not only in the last sea ice-covered and pristine regions in the AO but also in the SO [106].

### 4.2. Metric suitability for characterizing offshore polar soundscapes

#### 4.2.1. Dominant characteristics

The PSD and the SPD are two metrics that are already widely applied and constitute a fundamental part of describing ocean soundscapes (e.g. [55–58]). In our study, the visual representation of the ambient sound in the context of the PSD along the SPD proved useful for identifying regional and seasonal salient sound sources (biophonic, geophonic and anthropophonic) and for visualizing seasonal soundscape characteristics. This seasonal visualization was valuable for understanding and interpreting the complexity of the diversity metrics. Moreover, it indicated that the set of selected metrics performed well in capturing seasonal soundscape characteristics. We, therefore, recommend the use of the PSD and SPD for visual investigation and interpretation of not only ambient sound levels but also of trends in the variability of spectro-temporal intensity distribution over large spatiotemporal scales.

#### 4.2.2. Geophonic characteristics

Broadband wind-mediated and cryonic sounds affected SPLs (63, 125, 250, 500 Hz TOL bands and broadband SPL) and spectral uniformity (AEI, ADI, HI and BI) by increasing ambient noise levels and by lowering signal-to-noise ratios, which is consistent with previous studies (e.g. [7,10,30,81]). In both regions, the 500 Hz TOL proved to be the most robust indicator of broadband wind-mediated and cryonic sounds and may hold promise as a monitoring standard for changes in environmental conditions. In addition, the sensitivity of the ACI to cracking cryonic sounds could make this measure a valuable tool for studying changes in sea ice stability. Overall, the sensitivity of these intensity and diversity metrics to wind-mediated and cryonic sounds could therefore serve as a useful measure for studying changes in sea ice stability, sea ice patterns or increasing open water periods.

#### 4.2.3. Biophonic characteristics

At the FS station, the weak acoustic presence of blue and fin whales was only indicated by small peaks at 18 and 20 Hz in the lower percentiles (1%–25%) of the PSD (figure 2). The respective TOL band did not properly reflect the presence of these species, owing to acoustic masking by flow noise and airgun noise. The sparse acoustic presence of sperm whales increased the ACI but did not affect the overall temporal heterogeneity of the soundscape.

At the WS station, the year-round fin and Antarctic blue whale choruses governed seasonal variation in ambient sound levels in the 25 Hz TOL band. In addition, peaks in the 63 Hz TOL band in the austral summer were in some cases indicative of Antarctic blue whale D-calls. During sea ice-covered conditions, the 125 Hz band was especially sensitive to intensity fluctuations in Antarctic minke whale choruses, while fluctuations in the 250 and 500 Hz TOL band in the course of austral spring were indicative of intensity variation in the leopard and crabeater seal choruses. Occasionally, high-intensity calls of Antarctic minke whales were also indicated by an increase in the ACI. Moreover, corresponding to previous studies (e.g. [68,104,107]), the AEI, ADI and HI indicated higher spectral uniformity for high call rates and rich acoustic biodiversity as the signal-to-noise ratio decreased owing to chorusing and more spectral niches becoming occupied. Siddagangaiah *et al*. [72] reported an increase in the ADI during fish chorusing, with harmonics occupying multiple frequency bands saturating the spectral acoustic space, similar to the call characteristics of Antarctic minke whales and the crabeater seals. Conversely, the presence of single high-intensity choruses such as fin and Antarctic blue whale choruses in the WS can decrease spectral uniformity. This observation aligns with other studies reporting a drop in the HI when chorus intensity increases and spectral uniformity is no longer given, as energy becomes concentrated into narrow bands [107,108]. This behaviour of the indices might be the key to the differentiation in soundscape characteristics between austral summer at the WS station and the FS station. In combination with the other diversity metrics, the BI could therefore be suitable as an indicator of soundscape patterns dominated by biophony forming spectral bands. This suggests that the diversity metrics along the TOL bands can function to monitor the influence of biophonic sources on shaping the characteristics and potential climatic-induced changes of the SO soundscape over large spatiotemporal scales.

#### 4.2.4. Anthropophonic characteristics

We found that the TOL bands recommended by the MSFD [25] effectively captured the impact of airgun noise on sound levels in the FS. Among the two TOL bands recommended by Merchant *et al.* [26], the 250 Hz band appeared to be most indicative of ship noise while the 500 Hz TOL band was strongly influenced by wind-mediated noise in the FS. To our knowledge, our study is the first to apply the TOL bands recommended by the MSFD [25] and Merchant *et al.* [26] to characterize ambient sound levels for the Atlantic sector of the SO. However, these TOL bands mainly reflected the seasonal vocal activity of marine mammals (§4.2.3) and environmental sounds (§4.2.3), instead of shipping noise as envisioned by the MSFD recommendations and Merchant *et al.* [26]. Without critical review or previous knowledge of the soundscape, these metrics would have characterized this area as strongly impacted by ship noise during periods with sea ice cover. However, in austral summer, the 63 and 125 Hz TOL bands were able to indicate airgun noise. Therefore, these bands might still be effective for monitoring anthropogenic noise in sea ice-free regions of the SO. Our results, therefore, call for caution in defining and applying global standard metrics. For the SO, other bands with less marine mammal interference would need to be selected for monitoring current global shipping noise.

#### 4.2.5. Identifying spatiotemporal soundscape patterns

In this study, metrics describing ambient sound levels (500, 250 and 63 Hz TOL band and broadband SPL) and spectral variability (AEI, ADI and HI) were most important in differentiating between seasonal and regional soundscape characteristics. The great importance of ambient sound levels is likely owing to seasonal variation in ambient sound levels at the WS station governed by the seasonality of marine mammal vocal activity and the dampening effect of sea ice cover. Our results in regards to the diversity metrics align with the study of Williams *et al*. [36] and Roca & Van Opzeeland [39] who reported the HI as one of the best-performing indices in differentiating between different marine habitat types (degraded, healthy, polar offshore and polar onshore). In contrast to our findings, these two studies found that the AEI and ADI were less important in discriminating between sites, while these studies considered the ACI and BI as most important [36,39]. Roca & Van Opzeeland [39] included soundscape observations from the SO with a higher abundance of Weddell seal calls, which might have resulted in a higher ACI variability among polar onshore and pelagic soundscapes. Consequently, the ACI might not have performed as well in our study for differentiating between seasonal and regional soundscape characteristics as both stations carried low or similar amounts of signals that the ACI responds to. This emphasizes that the importance of different metrics to differentiate soundscapes might vary with the spatiotemporal scale of the dataset in question (e.g. see also [32]), and a combination of multiple metrics provides a more robust monitoring standard for soundscapes spanning large spatial and temporal scales.

In our study, we used hourly mean values of EAMs to differentiate soundscape characteristics, which might have smoothed out more subtle soundscape variability but which provided a good overall representation of the overall spatiotemporal soundscape variability. However, we did not account for variability within soundscape properties, for example, the 95% confidence interval [40] or standard deviation [32], which has been shown to capture more subtle soundscape characteristics and improved characterization among different habitats [32]. Consequently, the interquartile range (25th to 75th percentile), which we used to indicate the intra-seasonal variability of ambient sound in at the FS station, might also be useful to include more subtle soundscape variation in the characterization of soundscapes.

Here, we focused on diversity metrics that emphasized spectral uniformity versus heterogeneity (AEI, ADI, HI and BI) and included lower-frequency TOL bands (maximum 500 Hz TOL band). The application of higher-frequency TOL bands will permit capturing additional local sound sources, and hence could improve soundscape characterization by including the full spectrum. Furthermore, including diversity metrics that emphasize temporal heterogeneity, such as temporal uniformity (Ht; [69]), or time-lagged autocorrelation of SPLs for quantifying periodicity [40], will likely also enhance the overall characterization of soundscapes. Wilford *et al*. [40] suggested incorporating further soundscape properties such as impulsiveness and recommended using kurtosis as a metric. They further applied the D-index, which supposedly better reflects the spectro-temporal uniformity in marine soundscapes compared with the HI [40]. In our study, however, we focused mainly on a suite of diversity metrics that have already been commonly applied (e.g. [29–31]). Nevertheless, Wilford *et al*. [40] recommended that standard metrics as well as other metric combinations are needed to determine the best set of EAMs to explore overall global soundscape characteristics and quantify the acoustic states.

## 5. Conclusions

Our results suggest that a combination of ecoacoustic intensity and diversity metrics is useful for investigating spatiotemporal soundscape characteristics in polar offshore regions in relation to anthropogenic noise and environmental conditions in order to assess the *status quo* of the marine acoustic environment. Our study uncovered differences in annual soundscape characteristics of our recording site in the Arctic FS strongly impacted by anthropogenic noise and the acoustically quasi-pristine Antarctic WS. It also provides a blueprint of a methodological approach to explore variations in polar acoustic offshore environments more holistically. Our results hence provide an essential baseline for further investigations of polar soundscape patterns on larger spatial and temporal scales, such as comparisons across the WS basin or the FS, which is particularly crucial in the light of climate change-induced alterations in soundscape regimes.

## Data Availability

The passive acoustic datasets analysed in this study are available through the PANGEA database: Thomisch *et al*. [46] (for data collected in the FS) and Thomisch *et al*. [47] (for data collected in the WS). The long-term spectrograms of the analysed recorders can be accessed via the Open Portal to Underwater Soundscapes (OPUS) accessible at (CC BY 4.0, AWI 2023 [52]). Supplementary material is available online [109].
